# Alternate levels versus all levels mini-plate fixation in C3-6 cervical laminoplasty: a retrospective comparative study

**DOI:** 10.1186/s12891-024-07638-0

**Published:** 2024-07-03

**Authors:** Feng-Yu Liu, Jin-He Yu, Li-Shuang Huo, De-Jing Meng, Kuan Lu, Zhao Liu, Liang Ren, Xian-Ze Sun

**Affiliations:** 1 Department of Spine Surgery, The Third Hospital of Shijiazhuang, No. 15 Tiyu Street, Shijiazhuang, 050000 China; 2 Department of Endocrinology, Shijiazhuang People’s Hospital, Shijiazhuang, 050000 China; 3 Emergency Follow‑up Department, Shijiazhuang Emergency Center, Shijiazhuang, 050000 China

**Keywords:** Alternate levels, Mini-plate fixation, Cervical, Laminoplasty

## Abstract

**Objective:**

The purpose of this study is to compare radiological and clinical outcomes between alternate levels (C4 and C6) and all levels mini-plate fixation in C3-6 unilateral open-door laminoplasty.

**Methods:**

Ninety-six patients who underwent C3-6 unilateral open-door laminoplasty with alternate levels mini-plate fixation (54 patients in group A) or all levels mini-plate fixation (42 patients in group B) between September 2014 and September 2019 were reviewed in this study. Radiologic and clinical outcomes were assessed. Clinical results included Visual Analogue Scale (VAS) of axial neck pain and Japanese Orthopedic Association (JOA) score. Radiographic results included cervical range of motion (ROM), cervical curvature index (CCI), and the spinal canal expansive parameters including open angle, anteroposterior diameter (APD), and Pavlov`s ratio.

**Results:**

There was no significant difference in VAS, JOA score, ROM, and CCI between two groups. There was no significant difference in canal expansion postoperatively between two groups. However, open angle, APD, and Pavlov`s ratio in group A decreased significantly during the follow-up. In group B, APD, Pavlov`s ratio, and open angle were maintained until the final follow-up. There was no hardware failure or lamina reclosure occurred in both groups during the follow-up. The mean cost of group B was higher than that of group A.

**Conclusions:**

Despite the differences in the maintenance of canal expansion, alternate levels mini-plate fixation can achieve similar clinical outcomes as all levels mini-plate fixation in C3-6 unilateral open-door laminoplasty. As evidenced in this study, we believe C3-6 laminoplasty with alternate levels (C4 and C6) mini-plate fixation is an economical, effective, and safe treatment method.

## Introduction

Unilateral open-door laminoplasty, first reported by Hirabayashi in 1977, is commonly used to treat multi-segmental cervical compressive myelopathy caused by ossification of the posterior longitudinal ligament or spondylotic disease [1, 2]. C3-7 laminoplasty is the most popular procedure though many variations of laminoplasty are developed [3]. However, it was reported that C3-6 laminoplasty with preservation of muscles attached to the C2 and C7 spinous processes reduces the incidence of prolonged postoperative axial neck pain and C2-C7 angle loss [4, 5]. C3-6 laminoplasty was adapted to multi-segmental cervical compressive myelopathy in our institution.

To provide enough space for the spinal cord to move away from anterior compression, adequate enlargement of the spinal canal is critical in unilateral open-door laminoplasty [1, 6]. Traditionally, sutures were used to fasten the spinous process to the facet joint capsule to keep the hinge open which was proved effective in resolving neurological symptoms. However, as the absence of rigid fixation, reclosure of lamina was reported during follow-up [7, 8]. Mini-plate fixation, first reported in 1996, is effective in preventing lamina closure by providing the lamina immediately rigid fixation [9]. Usually, the plate is applied at each level. As mini-plates are costlier, it is also used at alternate levels to reduce the cost for the patients [1, 6, 10–12]. We performed alternate levels(C4 and C6) fixation in C3-6 cervical laminoplasty for some patients.

To date, no comparative study on plates used at alternate levels(C4 and C6) or all levels in C3-6 cervical laminoplasty has been published. Whether they could achieve similar radiographic and clinical outcomes is still questionable. The purpose of this study is to compare radiological and clinical outcomes between alternate levels(C4 and C6) and all levels mini-plate fixation in C3-6 unilateral open-door laminoplasty.

## Materials and methods

Radiological imaging studies and medical records of patients who underwent C3-6 unilateral open-door laminoplasty between September 2014 and September 2019 in our institutional were retrospectively reviewed.

The inclusion criteria were as follows: (1) Diagnosis of ossification of posterior longitudinal ligament or cervical spondylotic myelopathy, (2) underwent C3-6 unilateral open-door laminoplasty as the primary surgery, and (3) mini-plate was used at alternate levels(C4 and C6) or all levels. The exclusion criteria were as follows: (1) fractures, tumors, and metabolic disorders, (2) structural spinal deformity, (3) concurrent anterior cervical spine surgery, and (4) previous history of cervical spine surgery.

Finally, a total of 96 eligible patients were enrolled in this study. Two alternate levels (C4 and C6) were fixed by mini-plates in 54 cases (group A). The other 42 patients were treated with all levels mini-plates fixation (group B).

### Surgical procedures

Before surgery, patients choose alternate levels mini-plate fixation or all levels mini-plate fixation according to their own financial situation. All operations were performed by the same spine surgeon. All bilateral muscles attached to the spinous processes of C2 and C7 were preserved. The interspinous ligaments from C3 to C6 were also preserved during dissection. For patients with C2/C3 spinal stenosis, the dome-like laminoplasty was performed to preserve the integrity of the C2 spinous processes [13]. For patients with C6/C7 stenosis, the partial laminectomy of C7 was performed to preserve the integrity of the C7 spinous processes. The side with more severe symptoms was the open side and the other side was the hinge side. After elevating the open side of the laminae, mini-plates with appropriate size were selected and screws were inserted in per plate. In Group A, mini-plates were installed on C4 and C6. The spinous processes of C3 and C5 were fixed to the articular capsule with sutures. Mini-plates were placed on C3, C4, C5, and C6 in Group B. All patients were treated with a rigid collar for four weeks postoperatively [14].

### Clinical evaluation

Visual Analogue Scale (VAS) of axial neck pain and Japanese Orthopedic Association (JOA) score system were used to evaluate the clinical outcome. The neurological recovery rate was calculated as (postoperative JOA score-preoperative score)/(17-preoperative score)*100% [6, 10]. Operation time, blood loss, hospital stay, and cost were reviewed for each case. Complications such as axial symptom, C5 palsy, cerebrospinal fluid leakage, hardware failure, and lamina reclosure were recorded until the final follow-up for all patients.

### Radiologic evaluation

**Fig. 1:**
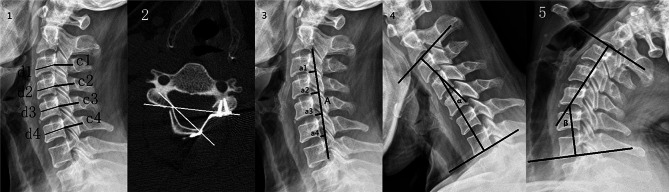
(**1**) Mean anterioposterior diameter = (c1 + c2 + c3 + c4)/4. Mean Pavlov`s ratio = (c1/d1 + c2/d2 + c3/d3 + c4/d4)/4. (**2**) Measurement of open angle on CT image. (**3**) Lateral radiograph showing evaluation of the cervical curvature index (CCI) with the Ishihara method: CCI=(a1 + a2 + a3 + a4)/A. (**4, 5**) The cervical range of motion (ROM) was measured on the flexion-extension radiograph. ROM = α + β

To assess radiographic changes, we reviewed cervical X-ray and three-dimensional CT at preoperative, postoperative, 3 months, 6months, 1 year, and final follow-up. Cervical range of motion (ROM) was evaluated on the flexion and extension radiograph. As described by Ishihara, cervical curvature index (CCI) was used to evaluate cervical lordosis [15]. The open angle, Pavlov’s ratio, and mean anterioposterior diameter (APD) were used to assess spinal canal expansion (Fig. 1). The criteria of reclosure is a 10% decrease in the APD, a 10% decrease in Pavlov’s ratio, and a decrease of 10◦ in open angle [16, 17]. All radiological measurements were made by two authors and the values were averaged.

### Statistical analysis

Data were imported into SPSS (version 21.0; SPSS Inc., Chicago, Illinois, USA). Normal distribution values were expressed as means ± standard deviation and were compared using an independent t test. Non-normal distribution values were presented as median and inter-quartile ranges and were compared by Mann-Whitney U test. Categorical variables were compared by Chi-square test. A *P* value of less than 0.05 was considered statistically significant.

## Results

### Patient characteristics

A total of 96 patients who matched the criteria were included in this study. Fifty-four patients who underwent alternate levels plate fixation (C4 and C6) were subdivided in group A, including 10 females and 44 males, with a mean age of 59.1 ± 9.8 years. The other 42 patients who underwent all levels plate fixation were subdivided in group B, including 9 females and 33 males, with a mean age of 58.7 ± 9.7 years. The mean follow-up time for two groups were 57.0(49.8, 65.0) months and 53.5(48.0, 61.0) months respectively. Duration of symptom for two groups were 8.0(3.0, 13.5) months and 8.0(4.5, 18.0) months respectively. Hospital stay for two groups were 14.8 ± 2.9 days and 15.0 ± 2.5 days respectively. Operation time for two groups were 143.2 ± 34.7 min and 150.6 ± 32.5 min respectively. Blood loss for two groups were 267.6 ± 66.7 ml and 282.1 ± 68.0 ml respectively. There was no significant difference in age, gender, duration of symptom, follow-up time, blood loss, operation time, and hospital stay between two groups. However, the mean cost in group B ($10904.6 ± 126.6) was higher than group A ($6436.6 ± 391.8) (*P* < 0.001) (Table 1).

**Table 1:** Patient characteristics

Characteristics	Group A	Group B	*P*
Number of patients	54	42	
Gender (male: female)	44:10	33:9	0.723
Age (years)	59.1 ± 9.8	58.7 ± 9.7	0.845
BMI (kg/m^2^)	26.6 ± 3.6	25.6 ± 2.7	0.137
Smoking	23	20	0.623
Duration of symptom (months)	8.0(3.0, 13.5)	8.0(4.5, 18.0)	0.736
Operation time (minutes)	143.2 ± 34.7	150.6 ± 32.5	0.292
Blood loss (ml)	267.6 ± 66.7	282.1 ± 68.0	0.296
Hospital stay(days)	14.8 ± 2.9	15.0 ± 2.5	0.657
Cost($)	6436.6 ± 391.8	10904.6 ± 126.6	< 0.001
Follow-up (month)	57.0(49.8, 65.0)	53.5(48.0, 61.0)	0.123
Complications (total)	11	9	0.899
C5 palsy	2	1	0.594
Axial pain	8	7	0.804
Cerebrospinal fluid leakage	1	1	0.686

Normal distribution values were expressed as means ± standard deviation and non-normal distribution values were presented as median and inter-quartile ranges

### Clinical results

The mean JOA score improved from 9.39 ± 2.27 preoperatively to 14.24 ± 1.94 (*P* < 0.05) at the final follow-up with a recovery rate of 67.03 ± 17.23 in group A and improved from 9.17 ± 2.05 preoperatively to 14.26 ± 2.11 (*P* < 0.05) at the final follow-up with a recovery rate of 68.29 ± 20.28 in group B. The mean VAS dropped from 2.50 ± 1.40 preoperatively to 1.19 ± 0.75 at the final follow-up in group A (*P* < 0.05) and from 2.60 ± 1.27 preoperatively to 1.00 ± 0.58 at the final follow-up in group B (*P* < 0.05). There was no significant difference in VAS, JOA score, and recovery rate of JOA between two groups (Table 2).

**Table 2:** Comparison of clinical outcomes between the two groups

	Group A	Group B	*P*
**Preoperative**			
JOA score	9.39 ± 2.27	9.17 ± 2.05	0.621
VAS score	2.50 ± 1.40	2.60 ± 1.27	0.731
**Final follow-up**			
JOA score	14.24 ± 1.94^*^	14.26 ± 2.11^*^	0.959
JOA recovery rate (%)	67.03 ± 17.23	68.29 ± 20.28	0.743
VAS score	1.19 ± 0.75^*^	1.00 ± 0.58^*^	0.179

^*^, *P* < 0.05 compared with the preoperative parameter

### Radiologic results

Compared with preoperation, cervical ROM and CCI had a significant decrease in both groups at the final follow-up. There was no significant difference in cervical ROM and CCI between the two groups (Table 3).

**Table 3:** Comparison of radiologic outcomes between the two groups

	Group A	Group B	*P*
**Preoperative**			
Curvature index(%)	21.03 ± 3.99	20.68 ± 3.54	0.660
Range of motion(°)	45.26 ± 4.61	44.81 ± 3.84	0.611
Anteroposterior diameter(mm)	14.06 ± 0.67	13.93 ± 0.63	0.325
Pavlov`s ratio	0.72 ± 0.06	0.71 ± 0.05	0.376
**Postoperative**			
Anteroposterior diameter	22.07 ± 0.68^*^	22.14 ± 0.65^*^	0.622
Pavlov`s ratio	0.99 ± 0.06^*^	1.01 ± 0.07^*^	0.262
Open angle(°)	34.28 ± 2.24	34.34 ± 2.06	0.892
**Final follow-up**			
Curvature index(%)	15.86 ± 3.81^*^	16.90 ± 3.55^*^	0.175
Range of motion(°)	31.28 ± 4.35^*^	32.02 ± 4.12^*^	0.396
Anteroposterior diameter(mm)	21.84 ± 0.67^*,#^	22.12 ± 0.66^*^	0.041
Pavlov`s ratio	0.96 ± 0.06^*,#^	1.00 ± 0.07^*^	0.005
Open angle(°)	33.18 ± 2.28^#^	34.30 ± 2.07	0.015

^*^, *P* < 0.05 compared with the preoperative parameter

^#^, *P* < 0.05 compared with the postoperative parameter

In both groups, the APD of the spinal canal and Pavlov’s ratio significantly increased after surgery. For group A, the mean APD increased from 14.06 ± 0.67 mm preoperatively to 22.07 ± 0.68 mm postoperatively and decreased to 21.84 ± 0.67 mm at the final follow-up. For group B, it increased from 13.93 ± 0.63 mm preoperatively to 22.14 ± 0.65 mm postoperatively and was maintained well at the final follow-up (22.12 ± 0.66 mm). There was similar result in Pavlov`s ratio. For group A, Pavlov`s ratio increased from 0.72 ± 0.06 preoperatively to 0.99 ± 0.06 postoperatively and decreased to 0.96 ± 0.06 at the final follow-up. For group B, it increased from 0.71 ± 0.05 preoperatively to 1.01 ± 0.07 postoperatively and was maintained well at the final follow-up (1.00 ± 0.07). As for open angle, there was no significant difference between two groups postoperatively (34.28 ± 2.24° vs. 34.34 ± 2.06°, *P* = 0.892). There was significant difference between two groups at final follow-up (33.18 ± 2.28° vs. 34.30 ± 2.07°, *P* = 0.015).

Radiologic outcomes in alternate levels mini-plate fixation group were assessed. For fixed levels, the mean APD increased from 14.07 ± 0.68 mm preoperatively to 22.07 ± 0.69 mm postoperatively and was maintained well at the final follow-up (22.04 ± 0.70 mm). For unfixed levels, the mean APD increased from 14.06 ± 0.68 mm preoperatively to 22.05 ± 0.68 mm postoperatively and decreased to 21.66 ± 0.65 mm at the final follow-up. There was similar result in Pavlov`s ratio. For fixed levels, Pavlov`s ratio increased from 0.72 ± 0.06 preoperatively to 0.99 ± 0.06 postoperatively and was maintained well at the final follow-up (0.98 ± 0.05). For unfixed levels, it increased from 0.72 ± 0.06 preoperatively to 1.00 ± 0.07 postoperatively and decreased to 0.95 ± 0.06 at the final follow-up. As for open angle, there was no significant difference between two groups postoperatively (34.28 ± 2.03° vs. 34.30 ± 2.17°, *P* = 0.945). There was significant difference between two groups at final follow-up (34.25 ± 2.05° vs. 32.83 ± 2.10°, *P* = 0.001). (Table 4)

**Table 4:** Radiologic outcomes in alternate levels mini-plate fixation group

	Fixed Levels	Unfixed Levels	*P*
**Preoperative**			
Anteroposterior diameter(mm)	14.07 ± 0.68	14.06 ± 0.68	0.921
Pavlov`s ratio	0.72 ± 0.06	0.72 ± 0.06	0.960
**Postoperative**			
Anteroposterior diameter	22.07 ± 0.69^*^	22.05 ± 0.68^*^	0.878
Pavlov`s ratio	0.99 ± 0.06^*^	1.00 ± 0.07^*^	0.465
Open angle(°)	34.28 ± 2.03	34.30 ± 2.17	0.945
**Final follow-up**			
Anteroposterior diameter(mm)	22.04 ± 0.70^*^	21.66 ± 0.65^*,#^	0.004
Pavlov`s ratio	0.98 ± 0.05^*^	0.95 ± 0.06^*,#^	0.007
Open angle(°)	34.25 ± 2.05	32.83 ± 2.10^#^	0.001

^*^, *P* < 0.05 compared with the preoperative parameter

^#^, *P* < 0.05 compared with the postoperative parameter

### Complications

A total of three patients reported C5 palsy (2 in group A and 1 in group B). All patients recovered completely within three months of conservative treatment. Axial pain was observed in fifteen patients (8 in group A and 7 in group B). All patients relieved within three months of oral analgesics. Two patients presented cerebrospinal fluid leakage (1 in group A and 1 in group B). The drainage tube was removed seven days after operation and the incision of drainage tube was sutured. Both patients recovered without sequela. There was no significant difference between the two groups in the rates of axial pain (*P* = 0.804), C5 palsy (*P* = 0.594), and cerebrospinal fluid leakage (*P* = 0.686).

There was no hardware failure or lamina reclosure occurred during the follow-up. All patients demonstrated bony fusion on the hinge side.

## Discussion

Unilateral open-door laminoplasty has become an effective surgical technique for patients with multilevel cervical compressive myelopathy [6]. It has been considered easy and safe by enlarging the spinal canal and providing sufficient space to keep the spinal cord away from anterior compression [11].

Traditionally, the range of laminoplasty was C3-7. However, the spinal cord is rarely affected at C6/C7 because antero-posterior canal stenosis is rare at C6/C7 and range of motion at C6/C7 is small [3]. To preserve the C7 spinous process and lamina, N Hosono et al. prospectively performed C3-6 laminoplasty. Compared with C3-7 laminoplasty, C3-6 laminoplasty with shorter operative time, lower incidence of axial neck pain, and smaller operative wound [3]. C3-6 laminoplasty, preserving the muscle attached to the spinous processes of C2 and C7, could maintain satisfactory neurologic improvement and reduce the incidence of prolonged postoperative axial neck pain and reduce postoperative loss of C2-7 angle [4]. In our institution, C3-6 unilateral open-door laminoplasty has been adapted to cervical myelopathy. To provide enough space for the spinal cord to move away from anterior compression, the dome-like laminoplasty technique was used for patients with C2/C3 stenosis and the partial laminectomy of C7 was performed for patients with C6/C7 stenosis.

It is critical to maintain the expansion of the spinal canal in a successful laminoplasty [10]. The traditional method for keeping the lamina opening is by tying the spinous process to the articular capsule with sutures. However, there is risk of spring-back phenomenon due to the insufficient strength of the sutures [6]. Recent years, in order to prevent lamina closure, mini-plate fixation was used to make the lamina fixed immediately [14]. Usually, the plate is applied at each level. As mini-plates are costlier, it is also used at alternating levels to reduce the cost for the patients [1, 6, 10–12]. Wu J et al. reported that mini-plate fixation in alternate levels (C4 and C6) may better preserve cervical lordosis than that in alternate levels (C3 and C5) [19]. The cephalic portion of the C3 lamina is overlapped and obstructed by the distal portion of the C2 lamina. Some additional exposure may be required to properly install the instrument during C3 plating. So, plating at C4/C6 may maintain more muscle insertion than plating at C3/C5 [19]. In our institution, mini-plates are used at alternate levels (C4 and C6) in C3-6 laminoplasty based on patients’ independent choice. During operation, the spinous processes of C3 and C5 were fixed to the articular capsule with sutures and the interspinous ligaments from C3 to C6 were also preserved during dissection. The plating segments may provide sufficient support to uphold the non-plating segments to avoid laminar closure.

The purpose of this study is to determine whether alternate levels (C4 and C6) mini-plate fixation can achieve similar radiological and clinical outcomes as all levels mini-plate fixation in C3-6 unilateral open-door laminoplasty. In the present study, there was no significant difference in hospital stay, operation time, and blood loss between two groups. However, alternate levels mini-plate fixation could reduce the cost. We found that there are no signifcant diferences in canal expansion postoperatively between the two groups. However, open angle, APD, and Pavlov`s ratio decreased significantly in alternate levels mini-plate fixation group during the follow-up. In all levels mini-plate fixation group, APD, Pavlov`s ratio, and open angle were maintained till the final follow-up. The result showed that all levels mini-plate fixation was superior to alternate levels mini-plate fixation in maintaining canal expansion. However, there was no significant difference in VAS, JOA score, and recovery rate of JOA between two groups.

Chen et al. observed suture suspensory fixation in cervical laminoplasty and came to conclution that the parameters, such as Pavlov’s ratio, APD, open angles, and cross-sectional area, decreased along with time, mainly within 6 months after surgery. The lamina reclosure rate of suture group was 36.5% [20]. Lee et al. reported that opened laminae showed approximately 10% decrease in APD and opening angle after classic Hirabayashi open-door laminoplasty. They found that laminar closure may occur primarily within 6 months after surgery and the fusion of hinge side may prevent further lamina closure. They observed closure rates of 45.4%, 22.7%, and 44.7%, respectively based on three different closure criteria: 5° and 10° decreases in open angle and a 10% decrease in AP diameter [16]. In the present study, no reclosure of lamina was observed in any patient during follow-up. The result might mean that alternate levels mini-plate fixation is superior to sutures suspension fixation in maintaining the enlargement of the spinal canal. In alternate levels mini-plate fixation, the interspinous ligaments and the ligamentum favum were preserved, the plating segments provide sufficient support to uphold the non-plating segments.

Several literature also compared clinical and radiological outcomes between alternate levels and all levels mini-plate fixation in cervical laminoplasty. Wang et al. reported that patients with alternate levels of mini-plate fixation showed lower JOA recovery rate, smaller APD and open angle compared with all levels mini-plate fixation [10]. In their study, plates were used at alternate levels (C3, C5 and C7). There was no suture between the spinous process and the articular capsule at C4 and C6, which may explain their results. In contrast, Zhang et al. observed that, although the open angle in the alternative-level group was significantly reduced during follow-up, satisfactory clinical outcomes were achieved with both alternative-level (C3, C5, and C7) and full-level (C3-C7) fixation [6]. According to Zhang et al., the open angle loss might be due to the following two reasons: (1) the measurement of open angle may also be affected by hyperosteogeny at the open end of the sutured segment; and (2) there may be a slight loss of open angle at suture segments before the fusion of the hinge side [6]. In this study, radiologic outcomes in alternate levels mini-plate fixation group were assessed. For fixed levels, the mean APD, Pavlov`s ratio, and open angle were maintained well at the final follow-up. Our result indicated that open angle, APD, and Pavlov`s ratio in alternative levels mini-plate fixation group decreased significantly during the follow-up. There may be a slight loss of canal expansion at suture segments before the fusion of the hinge side. However, no lamina reclosure was observed in any patient. For patients who underwent alternate levels (C4 and C6) mini-plate fixation laminoplasty, the spinous processes of C3 and C5 were fixed to the articular capsule with sutures and the interspinous ligaments from C3 to C6 were also preserved during dissection. The plating segments may provide sufficient support to uphold the non-plating segments to avoid laminar closure. There was no difference in clinical outcomes between the two groups at last follow-up. So, alternate levels mini-plate fixation (C4 and C6) in C3-C6 laminoplasty might be a safe, effective, and economical fixation method.

In the context of China’s new medical reform, the price of internal fixation has decreased significantly. So it may not save as much money as it used to. But we think our research has some value. First, even if the price of internal fixation is reduced, alternate levels mini-plate fixation may reduce some of the burden on patients. Second, price of internal fixation may not have declined in other countries, and this study is a reference for patients in other countries. Third, our results show that alternate levels mini-plate fixation can achieve the same clinical outcomes, and therefore alternate levels mini-plate fixation is feasible. This can reduce some surgical procedures, because fewer surgical procedures can reduce the risk of error.

The loss of ROM and CCI were seen in our result. There was no significant difference in ROM and CCI between two groups. The main reasons may be the destruction of posterior cervical structure and the restriction of cervical spine after operation [6]. In our study, we performed C3-C6 laminoplasty preserving the muscle attached to the spinous processes of C2 and C7 and encouraged patients to exercise four weeks after surgery. It may help to preserve ROM and CCI. The common complications after laminoplasty were C5 palsy and axial pain. Our result showed no significant difference in the incidence of C5 palsy and axial pain between the two groups.

Some limitations should be noted. As our study is a retrospective single-institution study, randomized controlled studies are needed to confirm these results. In addition, there was substantial selection bias because patients choose alternate levels mini-plate fixation or all levels mini-plate fixation according to their own financial situation.

## Conclusion

Despite the differences in the maintenance of canal expansion, alternate levels mini-plate fixation can achieve similar clinical outcomes as all levels mini-plate fixation in C3-6 unilateral open-door laminoplasty. As evidenced in this study, we believe C3-6 laminoplasty with alternate levels (C4 and C6) mini-plate fixation is an economical, effective, and safe treatment method.

## Data Availability

The datasets used and/or analysed during the current study are available from the corresponding author on reasonable request.

## Abbreviations

APD: Anteroposterior diameter.
CCI: Cervical curvature index
JOA: Japanese Orthopedic Association
ROM: Range of motion
VAS: Visual Analogue Scale
